# Physiological and transcriptomic analyses reveal the molecular networks of responses induced by exogenous trehalose in plant

**DOI:** 10.1371/journal.pone.0217204

**Published:** 2019-05-22

**Authors:** Yongchun Shi, Hui Sun, Xiaoran Wang, Weihuan Jin, Qianyi Chen, Zhengdong Yuan, Haidong Yu

**Affiliations:** College of Life Sciences, Henan Agricultural University, Zhengzhou, Henan, China; Iowa State University, UNITED STATES

## Abstract

It is well known that exogenous trehalose can improve resistances of plants to some abiotic and biotic stresses. Nonetheless, information respecting the molecular responses of tobacco leaves to Tre treatment is limited. Here we show that exogenous Tre can rapidly reduce stomatal aperture, up-regulate NADPH oxidase genes and increase O_2_^•-^andH_2_O_2_ on tobacco leaves at 2 h after treatment. We further demonstrated that imidazole and DPI, inhibitors of NADPH oxidase, can promote recovery of stomatal aperture of tobacco leaves upon trehalose treatment. Exogenous trehalose increased tobacco leaf resistance to tobacco mosaic disease significantly in a concentration-dependent way. To elucidate the molecular mechanisms in response to exogenous trehalose, the transcriptomic responses of tobacco leaves with 10 (low concentration) or 50 (high concentration) mM of trehalose treatment at 2 or 24h were investigated through RNA-seq approach. In total, 1288 differentially expressed genes (DEGs) were found with different conditions of trehalose treatments relative to control. Among them, 1075 (83.5%) were triggered by low concentration of trehalose (10mM), indicating that low concentration of Tre is a better elicitor. Functional annotations with KEGG pathway analysis revealed that the DEGs are involved in metabolic pathway, biosynthesis of secondary metabolites, plant hormone signal transduction, plant-pathogen interaction, protein processing in ER, flavonoid synthesis and circadian rhythm and so on. The protein-protein interaction networks generated from the core DEGs regulated by all conditions strikingly revealed that eight proteins, including ClpB1, HSP70, DnaJB1-like protein, universal stress protein (USP) A-like protein, two FTSH6 proteins, GolS1-like protein and chloroplastics HSP, play a core role in responses to exogenous trehalose in tobacco leaves. Our data suggest that trehalose triggers a signal transduction pathway which involves calcium and ROS-mediated signalings. These core components could lead to partial resistance or tolerance to abiotic and biotic stresses. Moreover, 19 DEGs were chosen for analysis of quantitative real-time polymerase chain reaction (qRT-PCR). The qRT-PCR for the 19 candidate genes coincided with the DEGs identified via the RNA-seq analysis, sustaining the reliability of our RNA-seq data.

## Introduction

Trehalose (Tre), as a non-reducing disaccharide, is formed by two α-glucose units linked through α, α-1,1-glucosidic bond (α-D-glucopyranosyl-[1,1]-α-D-glucopyranoside). Tre biosynthesis and signaling in vivo have been investigated extensively in many different organisms, including bacteria, yeast, fungi, insects, plants and animals[1, 2]. Even though alternative pathways exist in different organisms, biosynthesis of Tre typically includes two steps. Trehalose-6-phosphate synthase (TPS) first catalyses the formation of trehalose-6-phosphate (T6P) from UDP-glucose and glucose-6-P, and trehalose-6-phosphate phosphatase (TPP) further convert T6P into Tre[3]. In vivo, Tre has been reported to protect the integrity of organelles and cells in some organisms against enviromental stresses[4–6]. T6P, as an intermediate metabolite of Tre biosynthesis, has been proved to function as a sensor for in vivo available sucrose, by this means regulating the responses of organism to the diverse environmental changes directly, which is reasonable as the components of Tre biosynthesis pathway, such as T6P, trehalose and their biosynthetic enzymes are part of an interactive correlation network including sugar and hormone signaling pathways, *etc*[7]. In plants, the components of Tre biosynthesis pathway not only influence growth and development, but also get involved in responses of both abiotic and biotic stresses[7–9]. Over-expressing yeast *TPS1* and *TPS2* in Arabidopsis can increase the resistances of the transgenic lines to abiotic stresses, including freezing, drought, salt and heat stress[10]. The *TPS1* transformants of sorghum exhibited tolerance to salt stress as well as higher root growth and biomass[11]. In rice, over-expression of *OsTPP1* confers rice tolerance to both salt and cold stresses[12], and *OsTPP7* was found as the genetic determinant in a major quantitative trait locus (QTL) for an aerobic germination tolerance[13]. Tre accumulated in Tripogonloliiformis can regulate autophagy that might further confer the plant desiccation tolerance[14].

Tre is one of naturally occurring substances produced by organisms, which is nontoxic to the environment. It showed elicitor and priming properties, and improved protection in plants against abiotic and biotic stresses.In wheat, exogenous Tre increases the resistance to the biotic stress caused by powdery mildew[15, 16]. In rice seedlings, Tre pretreatment is involved in protection against salt-induced oxidative damage through significantly enhanced level of antioxidant activity[17], and also elevated the endogenous Tre level and significantly withstood the toxicities of excessive copper on plant photosynthesis and development[18]. Under drought stress, a significant correlation has been found between exogenous application of Tre and drought tolerance, and foliar spray of Tre was the best way in improving antioxidant defense system in *Raphanus sativus L*. (radish) plants[19, 20]. A couple of studies have been done to investigate gene expression patterns in response to Tre treatment in Arabidopsis seedling cultured in liquid medium by DNA microarray[21, 22]. In tobacco, interestingly, exogenous Tre can effectively increase nitrogen metabolism, and promote tobacco growth under deficient nitrogen that restrict plant growth severely[23].

*N*. *tabacum* is the chief commercial crop among more than 70 species of tobacco known. Tobacco mosaic disease (TMV) is one of the major diseases of flue-cured tobacco. Studies have shown that pretreatment with exogenous Tre can enhance plant's tolerance to both abiotic and biotic stresses. However, whether Tre can enhance tobacco resistance to TMV has not yet been documented. In the present study, we found that exogenous Tre can enhance resistance of tobacco leaves to TMV in a concentration dependent way. To elucidate the molecular mechanism of the tobacco leaf in response to exogenous Tre treatment, firstly, the best concentrations and time-points of exogenous Tre treatment were determined by measuring a series of physiological responses of tobacco leaves to exogenous Tre, and combined with the resistant efficiency of tobacco to TMV affected by the different concentrations of Tre; secondly, the transcriptomic responses of tobacco treated with Tre were investigated using RNA-seq analysis. Our research provides a critical basis for understanding the precise mechanisms that occur in leaf tissues of the commercial crop *N*. *tabacum* in response to exogenous Tre treatment.

## Materials and methods

### Plant material, treatment with exogeneous Tre and inoculation with TMV

Tobacco seeds (*Nicotiana tabaccum* cv. k326 and *Nicotiana glutinosa*. *L*.) were germinated in petri dishes moistened filter paper. After 10 days, seedlings were transferred in compost (Petersfield Products, Leicester, UK) with no any other fertilizers in plastic pots (4 × 4 × 5 cm); 4–5 weeks later, plants were transferred to bigger plastic pots (diameter 20 cm; height 20 cm) containing the same compost. All plants used in this study were grown in a constant temperature greenhouse at 24°C with > 70% ambient humidity and 16 h light daily. Average photosynthetic photon flux density of 300μmol (photon) m^−2^ s^−1^ at the height of leaves employed for experiments. Tre treatment was tested for its activity of increasing N. glutinosa resistance against TMV using the half-leaf method (Necrotic local lesion assay in tobacco leaves by means of half leaves). Tre solutions (Amresco, USA) of 10 and 50 mM were freshly prepared before use in distillated water added with 0.025% (v/v) Citowett, a wetting agent, whereas the corresponding control contained distillated water with 0.025% (v/v) Citowett only. When tobacco had eight fully expanded true leaves healthily at the 8 leaves stage, leaves of the same age on different plants were selected for Tre treatment, usually they were the sixth and the seventh. Half of the whole leaf on both adaxial and abaxial surface was smeared 1 ml of distillated water as a control, and the other half was done with 1 ml of 10 mM or 50 mM Tre, respectively. For inoculation of TMV, the adaxial surface of each leaf was gently rubbed with 100μl of TMV in 10mM phosphate buffer (pH 7.0) at concentration of 10 μg/ml and Carborundum (silicon carbite) at 24h after Tre pretreatments. Numbers of local lesions on each half leaf were noted at 4 days after TMV inoculation. Fifteen replicates were performed for each Tre treatment and control. The rate of inhibition was calculated in accordance to the formula as follow:

$$Inhibition\;rate\;(\%)=\frac{Lesion\;number\;of\;control–Lesion\;number\;of\;Tre\;treatment}{Lesion\;number\;of\;control}\times 100$$

### Statistical data

To compare values from the raw data-number of lesions on each leaf infected by TMV and stomatal apertures of tobacco leaves treated with different concentration Tre, ANOVA was performed with the statistical software DPS Data Processing System software V15.10 [24]. The Student-Newman-Keuls (SNK) (P< 0.05) method was used to discriminate among the means.

### Histochemical detection of O_2_^•-^ and H_2_O_2_ accumulation in tobacco leaf

The histochemical detection of H_2_O_2_ and O_2_^•-^in tobacco leaf was performedaccording to Hernandez et al. [25] with minor modifications. NBT staining was used to detect *in situ* the production of O_2_^•-^. 25 mM K-HEPES [4-(2-hydroxyethyl)-1-piperazineethanesulfonicacid] buffer (pH 7.6) containing 0.1 mg mL^-1^ NBT was infiltrated into tobacco leaves directly by a vaccum, and incubated for 2 h at 25°C in the dark. Tobacco leaf was then rinsed for 10 min with 80% (v/v) alcohol at 70°C, and subsequently mounted in lactic acid/phenol/water [1:1:1, (v/v)] to eliminate the chlorophyllcompletely, andphotographed directly using camera.

In the case of H_2_O_2_, DAB (3,3ʹ-Diaminobenzidine) stainingwas used to detect *in situ* the production of H_2_O_2_. 50 mm Tris-acetate buffer (pH 5.0) containing 0.1 mg/mlDAB was infiltrated intotobacco leavesas above that were immersed into the same buffer for 24 h in the dark at 25°C. Controls were carried out and immersedinto the presence of 10 mm ascorbic acid.

### RNA-seq library preparation and illumina sequencing

Tobacco total RNAs from the leaf samples with Tre pretreatment were extracted using RNeasy Plant Mini Kit (Qiagen GmbH, Gemany) as stated in the manufacturer’s protocol. Firstly, RNA integrity and contamination was assessedin 1.0% agarose gel by electrophoresis. Further,the quality and concentration ofeach RNA sample were analyzedtaking advantage of an Agilent 2100 Bioanalyzer. The NEBNext Ultra RNA Library Prep Kit for Illumina (NEB, USA) was used for generation of sequencing librariesaccording to manufacturer’s recommendations, and the Agilent Bioanalyzer 2100 system was used fordetermination of library quality, which were then sequenced to generate 150 bp paired-end reads by 1 Gene Co. Ltd, Hangzhou, China using the Illumina HiSeq 4000 Platform.

### Functional annotation and enrichment pathway analysis of DEGs

In order to conduct the analysis of the raw RNA-Seq data, clean data (clean reads) were obtained by removing low quality reads (reads containing >20% of bases having Q value ≤15 or an ambiguous sequence content (ploy-N) more than 5% from raw data) by Trimmomatic (v0.30) [26]. The reference of tobacco genome and annotation files of gene model was obtained from the tobacco genome website to process the clean data with high quality (ftp://ftp.solgenomics.net/genomes/Nicotiana_tabacum/). Index of the reference genome was built using Bowtie2 (2.3.0) [27], and subsequently, the reads were blasted to the reference genome of tobacco using TopHat2 [28].

The gene transcript abundance in all 12 samples was calculated as fragments per kilo bases per million reads (FPKM) values[29]. The genes with FPKM<1 were filtered out before subsequent analysis. Differential expression of two conditions was analyzed using DESeq [30] that supplies statistical principles for assessing differential expression in RNA-seq data based on the negative binomial distribution. The Benjamini-Hochberg adjusted P values were used for controlling the false discovery rate (FDR). Gene with a very stringent cutoff, an adjusted FDR <0.05 and |log_2_Ratio|≥1 identified by DESeq, was classified as differentially expressed.

The Gene Ontology (GO) enrichment analyses of DEGs were performed using the topGO R package with an adjusted P-valueless less than 0.05 (http://www.geneontology.org/). EdgeR package was used for testing the statistical enrichment of DEGs in KEGG pathways (http://www.genome.jp/kegg/).

### qRT-PCR analysis for the DEGs

Tobacco total RNAs from leaf tissues sampledafter exogenous Trepretreatment were extracted using the RNAiso Plus kit with DNase I (Takara, Japan). First-strand cDNA was generated withthe oligo (dT) prime using the PrimeScript OneStep RT-PCR Kit Ver. 2 (Takara, RR055A). qRT-PCR experiment was performed usingthe CFX96 Touch™ Real-Time PCR Detection System (Bio-Rad Laboratories, Inc., USA) with the SYBR Green PCR Master Mix kit (PE-Applied Biosystems, USA).

## Results

### Pretreatment with exogenous Tre enhances resistance of tobacco leaf to TMV infection significantly

In this study, we took advantage of the half-leaf method to determine the optimal Tre concentration against TMV, We found were that both of 10 and 50 mM of Tre treatment before TMV infection can enhance resistance of tobacco to TMV infection significantly in the light of the numbers of local lesions on the leaves of *N*. *glutinosa* (Table 1 and S1 Data). The inhibition rates from 10 and 50 mM of Tre pretreatments against TMV were 17.1% and 36.23%, respectively, which are positively correlated with the concentration of exogenous Tre. Tre treatment during and after TMV infection also showed protection effects, butwas less efficient than a treatment before infection based on our preliminary results (data not shown).

**Table 1:** Protection of trehalose against TMV.

Trehalose/mM	Lesion number		Inhibition rate/%
	Control	The leaves with trehalose	
10	737	611	17.1
50		470	36.23

### The exogenous Tre as an effective elicitor increases superoxide anion (O_2_^•-^) and hydrogen peroxide (H_2_O_2_), reduces stomata aperture and activates the expression of *rbohD*/*rbohF*

**Fig 1:**
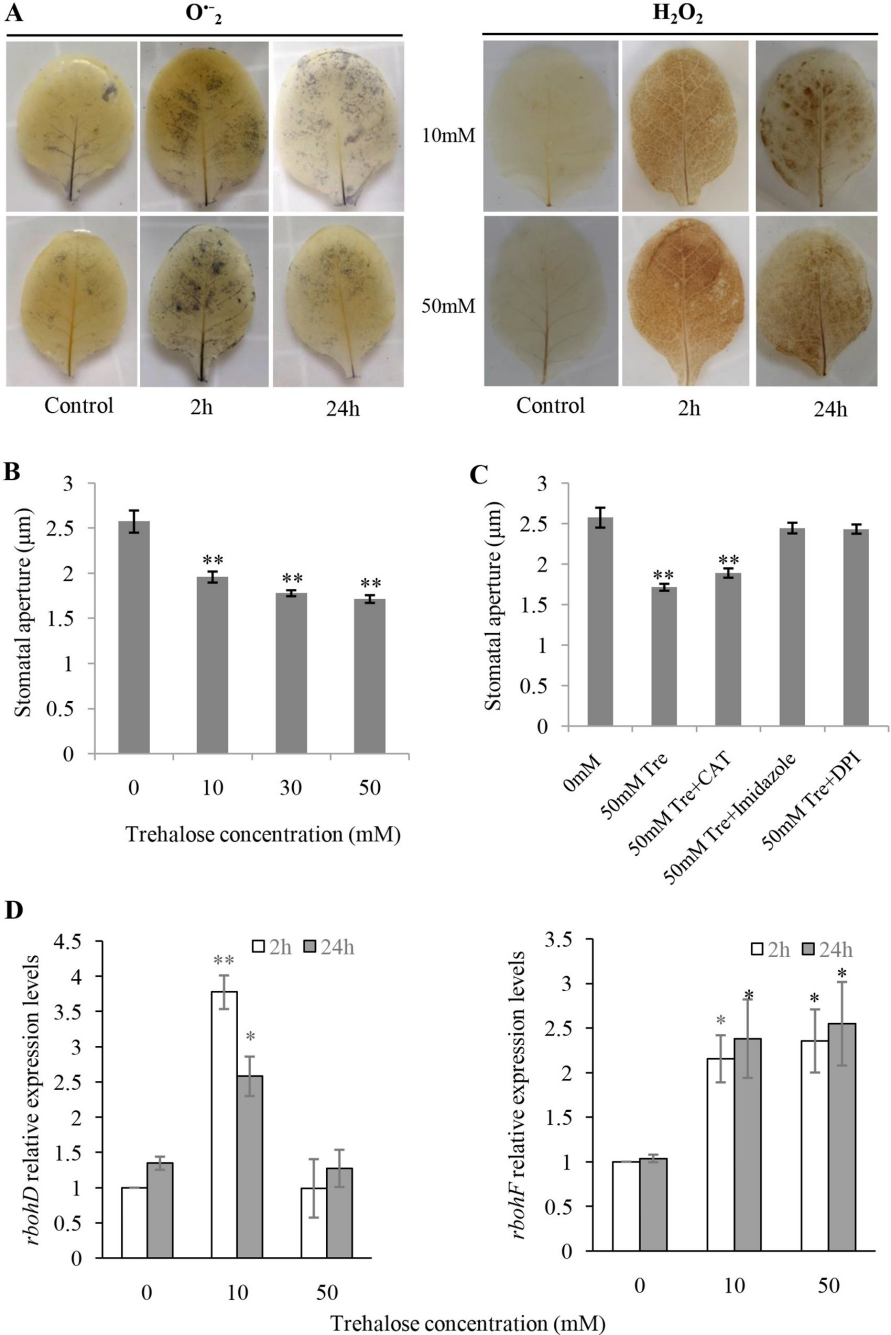
ROS content, stomatal aperture and enzyme expression profiles of tobacco leaves in response to exogenous trehalose. (**A**)Both superoxide ion (**O**^•-^_**2**_) and hydrogen peroxide (H_2_O_2_) were induced significantly at 2h and 24h after trehalose treatment; The effect of trehalose on stomatal aperture without (**B**) (n = 48) or with (**C**) (n≥22) CAT, imidazole and DPI;(**D**) The effect of trehalose on the transcript level of *rbohD* and *rbohF*.

Combined with the effect of Tre concentration on inhibition rate of TMV infection, a series of physiological phenotypes of tobacco leaves in response to exogenous Tre were characterized to identify the optimal time-points of Tre treatment for RNA-seq library construction. The sixth and seventh fully expanded true leaves of *N*. *tabaccum* at the 8 leaves stage were smeared with 2 ml of 0 (as a control), 10 and 50 mM of Tre on both sides, respectively, and the ROS species, O_2_^•-^ and H_2_O_2_, were monitored at 2h and 24 h after pretreatment separately as described in methods. NBT staining and DAB staining indicated that the production of both H_2_O_2_ and O_2_^•-^were significantly increased in tobacco leaves at 2 and 24 h after Tre treatment (Fig 1A). The stomatal aperture was also measured at 2h after the treatments with 0, 10, 30, and 50 mM Tre, respectively. After Tre treatment, the stomatal aperture of tobacco decreased significantly, and the percentage decreases were 23.9%, 30.9% and 33.4%, respectively (Fig 1B).

NADPH oxidases are generally membrane bound, and also named as respiratory burst oxidase homologues (RBOHs) that catalyze the reduction of molecular oxygen into O_2_^•-^ by transferring an electron, wherein NADPH acts as an electron donor and are the biological ROS factory and play a key role in the production of ROS in response to both abiotic and biotic stress in plants[31–33]. Based on that, some of the tobacco RBOHs might be activated by exogenous Tre, which is likely to be due to the induction of the stomata movement and the production of ROS in tobacco leaf. To test whether the tobacco RBOHs are involved in that procedure, we treated the tobacco leaves with 50 mM Tre combined with catalase (CAT) (Sigma-Aldrich, cat#: C9322), imidazole and DPI, respectively, as shown in Fig 1C. CAT is a catalase that clears H_2_O_2_ from cells. Imidazole and DPI, as inhibitor of NADPH oxidase, can bind to cytochrome B and flavoprotein in NADPH oxidase to hamper ROS production. Exogenous CAT treatment had little effect on Tre-induced reduction of stomatal aperture, while imidazole and DPI treatment blocked Tre-induced stomatal closure and returned to control levels of 95.02% and 94.52%, respectively (Fig 1C). In *N*. *tabacum*, three members of NADPH oxidases/RBOHs have been reported, and *NtrbohD* and *NtrbohF* are responsible for ROS production[34–36]. The expression profiles of *NtrbohD* and *NtrbohF* in response to exogenous Tre were determined by qRT-PCR. *NtrbohD* was up-regulated only by the treatment of 10 mM Tre at 2 and 24h significantly, however, *NtrbohF* was up-regulated in all the four conditions (10T2h, 50T2h, 10T24h and 50T24h) (Fig 1D).

### Sequencing overview and transcript identification

To investigate the molecular mechanism of the elicitor effect of exogenous Tre,cDNA samples were sequenced using the Illumina HiSeq 4000 platform. According to the resistance of tobacco leaves with Tre treatment to TMV infection and the physiological phenotypes above, the leaves treated with 10 (low concentration) and 50 (high concentration) mM of Tre were collected at two time-points, 2 (early stage) and 24h (late stage) for RNA-seq analysis with two replicates with a total of 12 samples, including controls and four different conditions/groups (combinations of two concentrations and two time-points). To simplify the description, we designated each two replicates of the tobacco leaves with 0 (control), 10 and 50 mM of T treatment for 2h or 24h as CT2h-R1 and -R2, CT24h-R1 and -R2, 10T2h-R1 and -R2, 50T2h-R1 and -R2, 10T24h-R1 and -R2, and 50T24h-R1 and -R2, respectively. Therefore, the four conditions/groups are named as 10T2h, 50T2h, 10T24h and 50T24h, respectively.

Sequencing results showed that more than 45 million reads for each sample were generatedfrom the 12 tobacco RNA libraries after the Illumina HiSeq 4000 sequencing (Table 2). Low-quality rRNA reads and adapters were removed, leading to theratio ofclean readsmore than 91% for each sample, which denotes that we had the sufficient sequencing depth for the transcriptome coverage in tobacco (Table 2). Observed percentages of reads mapped to the *N*.*tabaccum* genome per library were 79.4, 81.0, 86.1, 86.0, 86.2, 90.1, 82.6, 86.7, 85.7, 87.4, 83.6and 87.2%,respectively(Table 2), implying the RNA-seq data are sufficient and reliable for following analysis of bioinformatics.

**Table 2:** Summary of reads mapped to the *N*.*tabaccum* genome.

Sample ID	Total Reads	Clean Reads	Total Mapped Reads	MappedPairReads	Mapped Genes	Q20 (%)	GC (%)
10T2h_1	46,008,894	91.98%	79.4%	69.6%	36882	98.76	44.05
10T2h_2	45,054,250	92.47%	81.0%	69.2%	36941	98.82	44.02
10T24h_1	45,591,406	93.05%	86.1%	76.4%	37746	98.74	44.30
10T24h_2	48,229,638	92.84%	86.0%	75.6%	38065	98.69	44.13
50T2h_1	47,315,538	93.37%	86.2%	75.6%	37745	98.69	44.20
50T2h_2	45,769,886	94.83%	90.1%	81.1%	37724	98.69	43.91
50T24h_1	47,589,142	93.66%	82.6%	70.6%	37530	98.75	43.91
50T24h_2	48,076,528	92.68%	86.7%	76.2%	37449	98.66	44.02
C2h_1	47,337,734	96.51%	85.7%	77.4%	37947	98.82	44.05
C2h_2	46,996,730	94.57%	87.4%	78.3%	37615	98.75	44.02
C24h_1	45,862,902	93.35%	83.6%	75.3%	37486	98.86	44.30
C24h_2	46,758,318	94.31%	87.2%	78.7%	37807	98.81	44.13

### DEGs responding to exogenous Tre and qRT-PCR validation

As shown in Table 3, in the four comparisons of 10T2hvs C2h, 50T2hvs C2h, 10T24h vs C24h and 50T24hvsC24h, there were 242, 102, 833 and 111 DEGs indentified by DESeq, respectively. The maximum number of DEGs was in 10T24h vs C24h, which indicates low concentration of T (10mM) at late stage (24h) triggers more DEGs than the other three conditions. 110, 64, 579 and 56 were up-regulated, while 132, 38, 254 and 55 were down-regulated in the 10T2hvs C2h, 50T2hvs C2h, 10T24hvs C24h and 50T24h, respectively (Table 3).

**Table 3:** The DEGs in tobacco leaves responding to trehalose compared with control.

Group	Total	UP	Down
10T2h vs C2h	242	110	132
50T2h vs C2h	102	64	38
10T24h vs C24h	833	579	254
50T24h vs C24h	111	56	55

**Fig 2:**
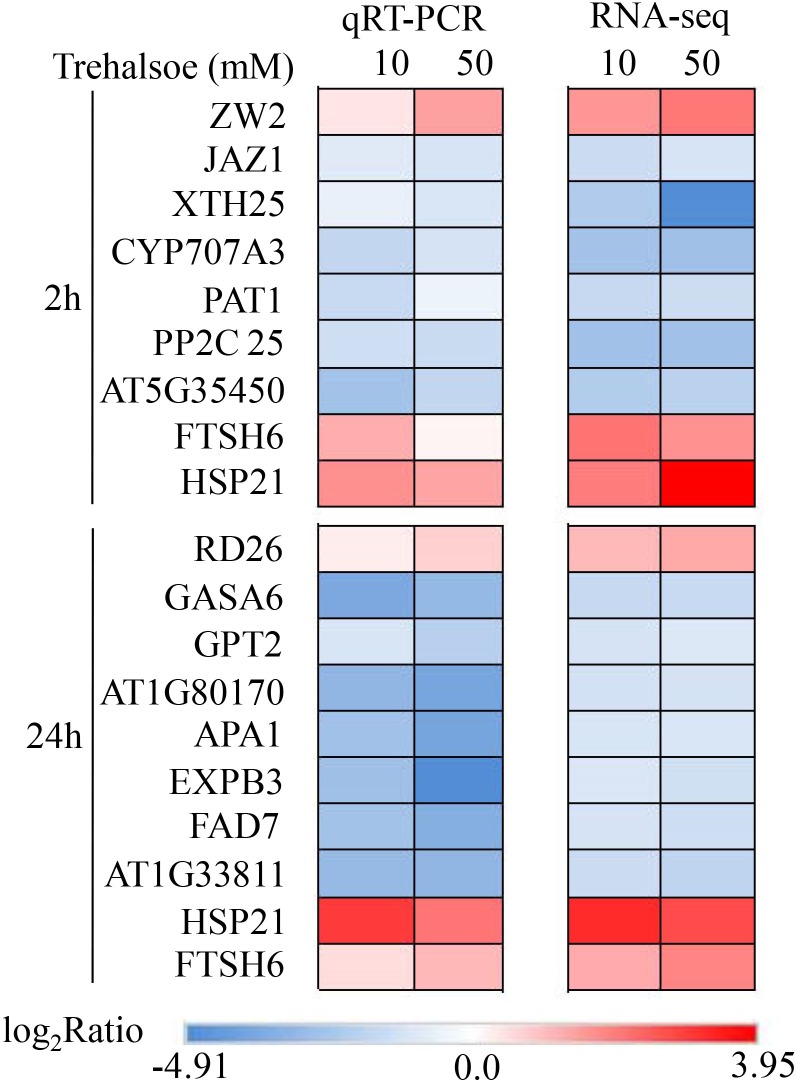
qRT-PCR validation of the DEGs selected from the four conditions (10T2h, 50T2h, 10T24h and 50T24h). The expression profiles were compared with the corresponding DEGs in RNA-seq data (R^2^ = 0.792, R^2^ = 0.873, R^2^ = 0.917, R^2^ = 0.871), respectively. R represents correlation coefficient.

To experimentally confirm that the DEGs obtained in this study were credible, expression profile of 19 selected DEGs, 2 (*FSH* and *HSP21*) of them up-regulated in all the four groups, were analyzed via qRT-PCR (Fig 2). The tobacco *Actin2* (GenBank accession No.EU938079.1) was chosen as a reference gene for qRT-PCRassay, and the2^-ΔΔCT^ method was used for calculation of relative expression levels of DEGs that were normalized to reference gene *Actin2* [37]. Three biological samples were prepared for each condition, and six conditions were included in Table 3. Three whole leaves except for main veins were collected as one independent biological sample. Two technical replicates for each sample were used for each DEG selected. The qPCR-RT results showed that the expression profiles of the DEGs selected were in line with those obtained from the Illumina sequencing analysis. As shown in Fig 2, comparison of qRT-PCR results with RNA-seq data showed high correlations (R2≥0.792 for each condition/group), confirming accountable RNA-seq analysis in the present study. These results indicated that the method used to determine DEGs in this study were valid. The primers used in qRT-PCR analysis were shown in S2 Data.

**Fig 3:**
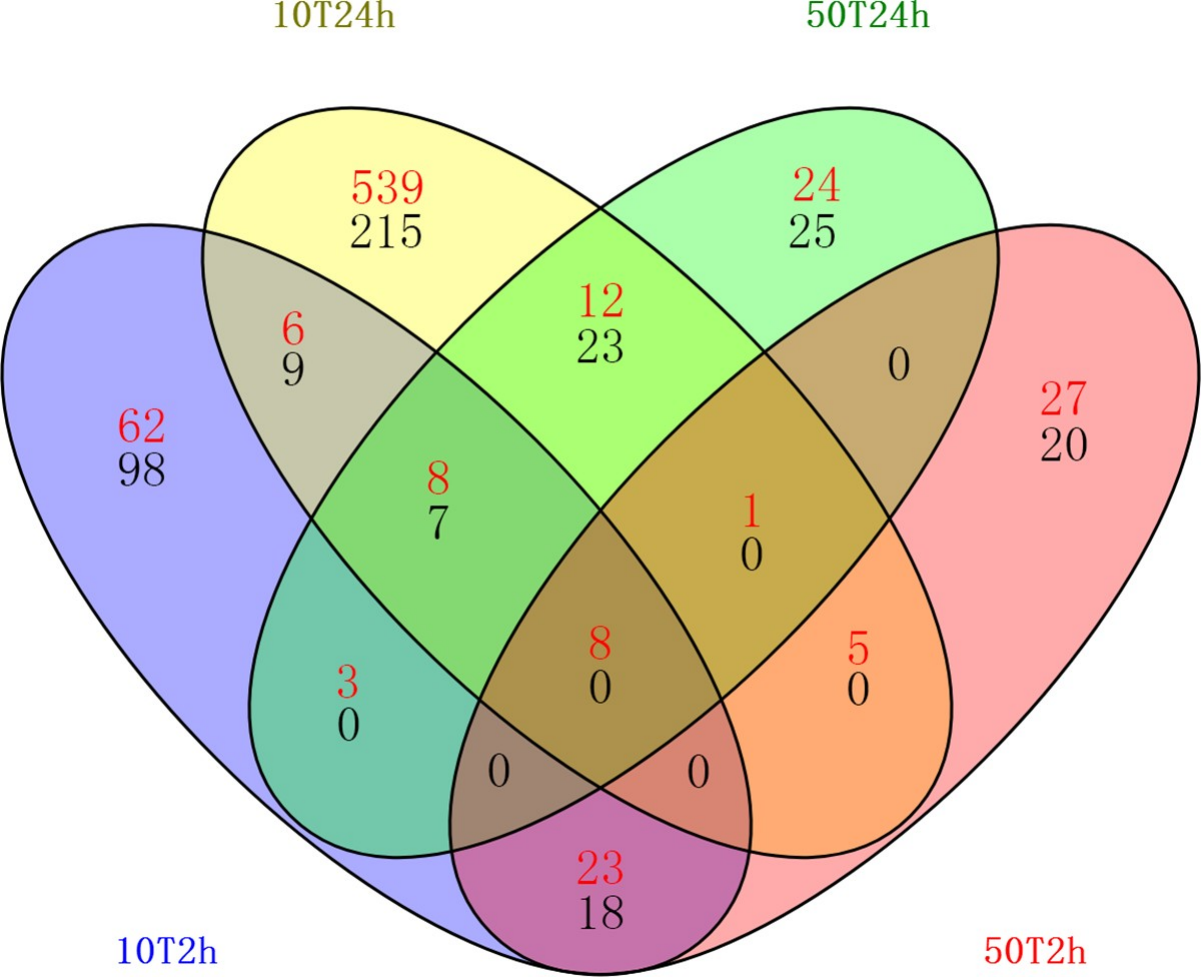
Venn diagram of the DEGs in different comparisons from the four conditions including 10T2h, 10T24h, 50T2h and 50T24h. The Numbers (Red: up-regulated genes; Black: down-regulated genes) indicate unique and common DEGs for the different comparisons.

A total of 1288 DEGs from all four conditions were further assorted by Venn analysis (Fig 3 and S3–S6 Data). The Numbers indicate unique and common DEGs for the different comparisons in the Venn diagram. From it, what we found was Tre affected the gene expressions of tobacco leaves in both a concentration-dependent manner and a time-specific manner. 929 (in response to 10 mM Tre) and 96 (in response to 50 mM Tre) DEGs regulated by effect of Tre concentration uniquely were confirmed, respectively (Fig 3, S7 and S8 Data), indicating that low concentration of Tre (10 mM) triggered much more genes expressed differentially than high concentration of Tre (50 mM) at both 2h and 24 h, and that was a much broader and more expensive response to 10 mM Tre. 160 unique DEGs were in 10T2h. Among them, 62 were up-regulated, and 98 were down-regulated. The majority of the DEGs regulated by 10 mM Tre were from 10T24h. Among them, 539 were up-regulated, while 215 were down-regulated. The DEGs also exhibited a time-specific expression pattern, a shift of numbers of DEGs from 2h to 24h was observed. Therefore, we compared and analyzed the DEGs in these two aspects.

### Functional classifications of the DEGs in response to Tre in a concentration-dependent manner by GO and KEGG pathway enrichment analysis

**Fig 4:**
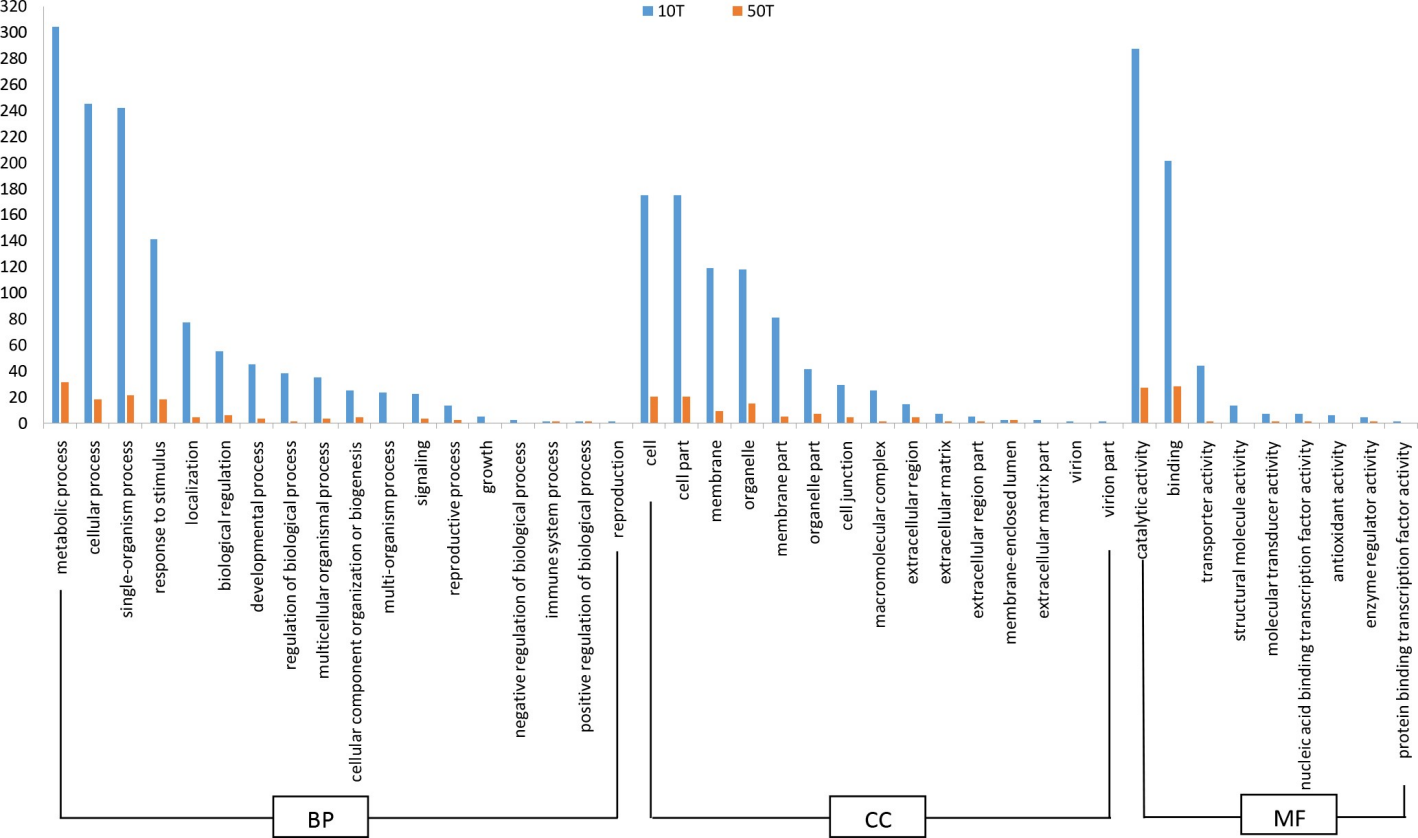
The comparison of the GO terms for the DEGs triggered by 10 mM and 50 mM of trehalose, respectively.

To uncover the similarities and differences of biological procedures in Tre-responsive transcriptomes between controls and the four different conditions, the 1288 DEGs from all four conditions were annotated and mapped to GO term and KEGG database, respectively. The Tre concentration comparison was performed in terms of the low (10mM) to high (50mM) concentration of Tre. Based on sequence homology, they were assigned to three ontologies of biological process (BP), cellular component (CC) and molecular function (MF) (Fig 4; S7 and S8 Data). The significant GO terms were very similar between the DEGs triggered by 10 mM Tre and 50 mM of Tre, and were mostly enriched in metabolic process, cellular process, single-organism process, and respond to stimulus in the ontology of BP. Most of the DEGs in the CC category were assigned to cell, cell part, membrane, organelle and membrane part. The top three GO terms in the MF category were catalytic activity, binding and transporter activity. The corresponding genes of these significant terms, therefore, might play important roles in response to abiotic and biotic stresses.

**Fig 5:**
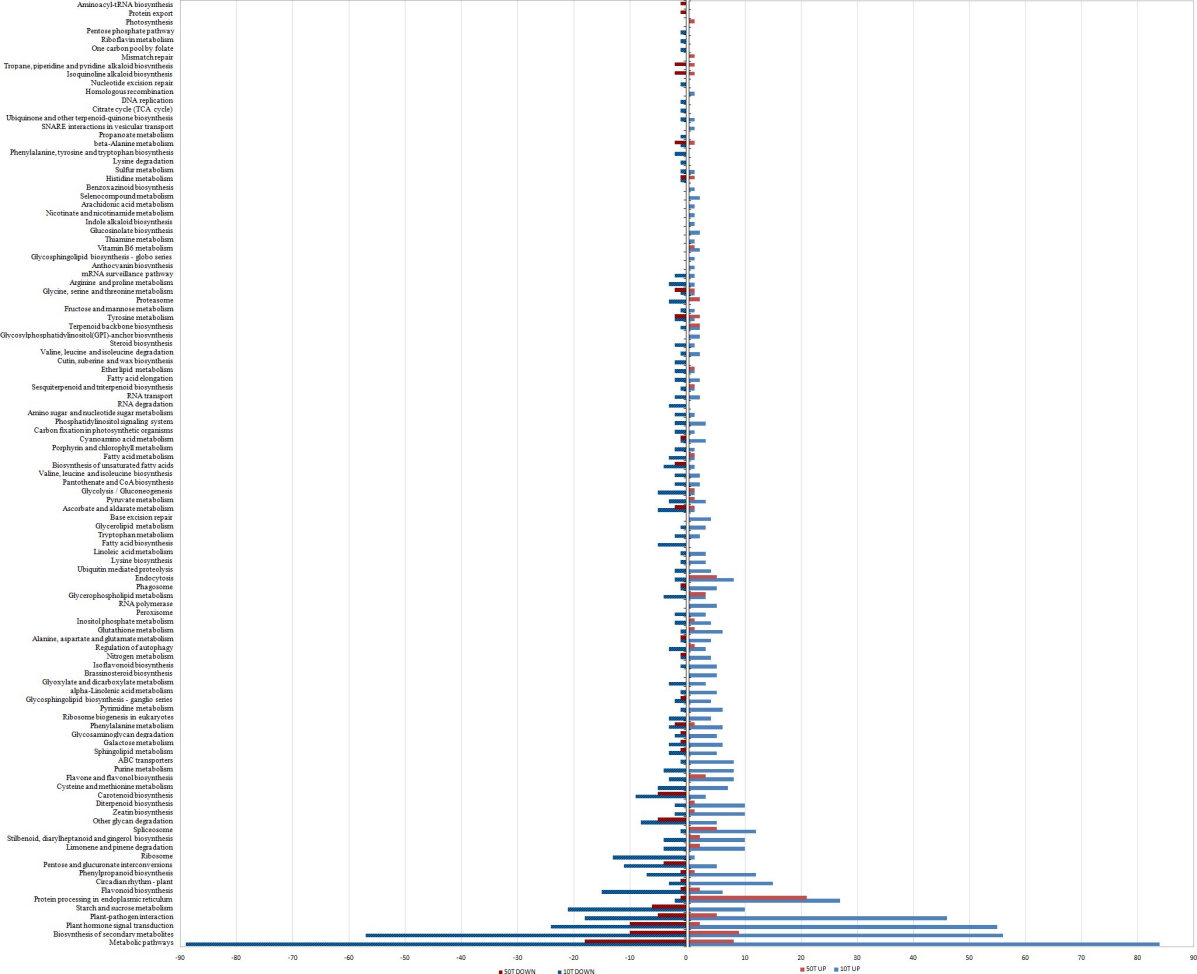
The Comparison of KEGG classification for the DEGs regulated by 10 mM and 50 mM of trehalose, respectively.

The DEGs from 10 mM and 50 mM of Tre treatment also had similar pattern in KEGG analysis. The 1288 total identified DEGs were assigned to 78 KEGG pathways (Fig 5, S7 and S8 Data) that were represented in Fig 5, and were largely enriched in biotic and abiotic stresses belong to metabolic pathway, biosynthesis of secondary metabolites, plant hormone signal transduction, plant-pathogen interaction, starch and sucrose metabolism, protein processing in ER, flavonoid biosynthesis, phenylpropanoid biosynthesis and pentose and glucuronate interconversion, *etc*. These results indicate that exogenous Tre can trigger a lot of stress-related genes in tobacco leaves, which might be involved in partial resistance to TMV. However, the amount of the DEGs in response to low concentration of Tre (10mM) were much more than those regulated by high concentration of Tre (50mM), their patterns were similar in different pathways though. For example, 173 DEGs triggered by 10 mM of Tre were enriched in metabolic pathway, in contrast, only 26 DEGs regulated by 50 mM of Tre treatment were found in the same pathway. However, in protein processing in ER, the amount (29) of DEGs triggered by 10 mM of Tre was similar to those (22) triggered by 50 mM of Tre. The overlap between them included 9 genes that were all increased in response to both of 10 and 50 mM of Tre treatment and encode heat shock proteins (Fig 5, S7 and S8 Data), which suggests that the concentration of exogenous Tre has a strong effect to the gene expression profiles of tobacco leaves.

### DEGs independent of Tre concentration in tobacco leaves relative to time-specific expression pattern

We further compared the differences and similarities of DEGs during the progression of Tre treatment as they might show the putative roles involved in resistance or susceptibility to abiotic and biotic stresses. At early time point (2h), 49 genes respond to Tre treatments free from effect of Tre concentration, in which 31 were up-regulated and 18 were down-regulated, respectively. At the later time point (24h), 35 genes respond to Tre treatments independent of effect of Tre concentration, where 12 were increased and 23 were decreased, respectively (Fig 3). Among these genes, 8 genes, named as core components in response to exogenous Tre, exhibited regulatory responses to all the four conditions, and were all up-regulated, including four heat shock protein (HSPs) genes (*HSP101*, *HSP90*, *HSP70T-2* and *HSP21*), two *FTHS6s*, one *GolS1*and one universal stress protein (USP) gene (S9 Data).

**Fig 6:**
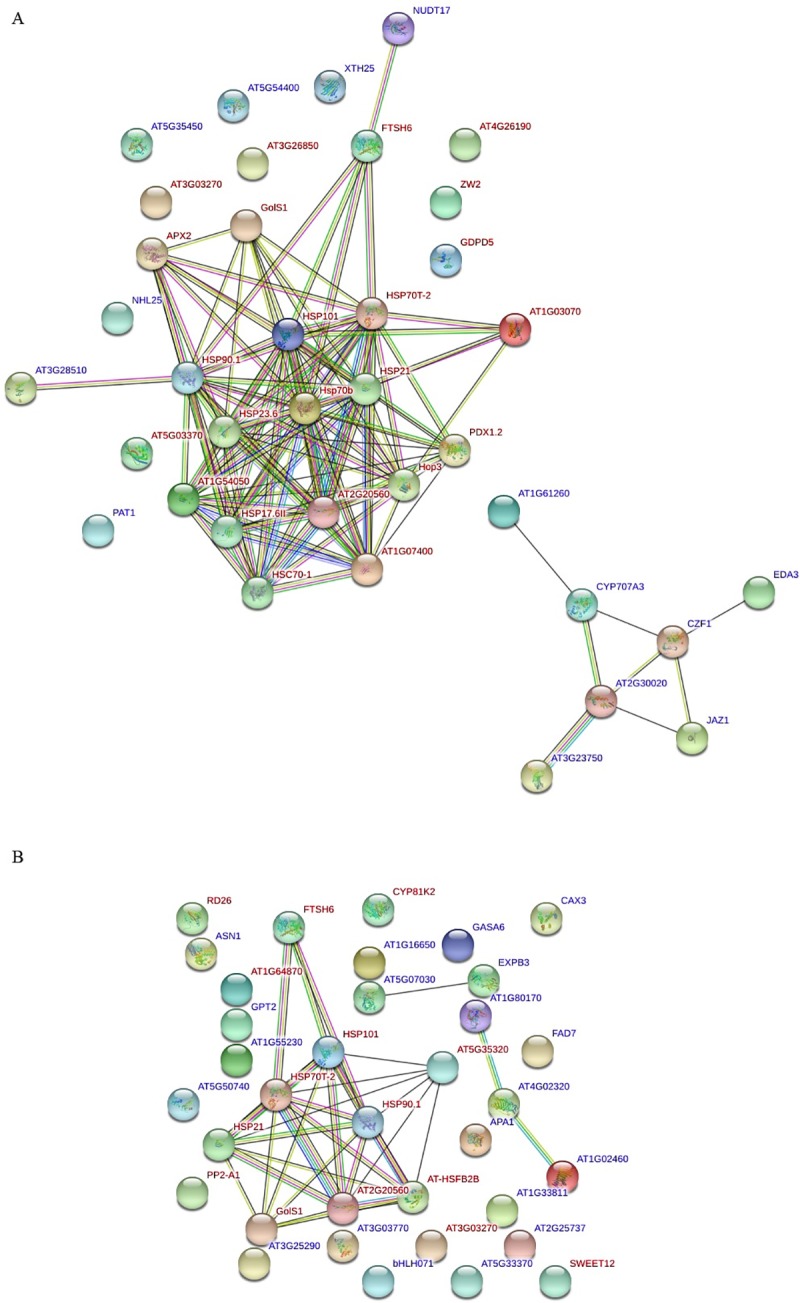
STRING network of trehalose-responsive genes at each time point (2h and 24h).

To elucidate the complex interaction of the differentially expressed proteins independent of effect of Tre concentration at each time point, the TAIR Arabidopsis gene codes of the DEGs were obtained via BLASTx (S10 and S11 Data), and subsequently imputed into STRING tool (www.string-db.org) to view protein-protein interaction networks (PPINs). The network of time point 2h indicates that Tre triggered a heat shock-like response at early stage (Fig 6A). Majority (31/49) of the DEGs were increased significantly, including the 8 core DEGs (S9 Data). Many of them, such as *APX2*, *GolS1*, *HSPs*, etc., are the targets of some key HSFs, like HSFA1 and HSFA2. HSFA2 might be responsible for their activations in tobacco leaves with Tre treatment since it has been reported that the peak of *HSFA2* expression is around 0.5-1h under heat stress [38, 39], which can explain *HSFA2* was not up-regulated significantly at 2h after Tre treatment in our RNA-seq data. In addition, jasmonate signaling pathway involved plant defense and diverse developmental pathways was clustered in PPIN of the 2h stage (Fig 6A), suggesting that Tre treatment can activate the pathway at early stage effectively. Down-regulation of JasmonateZim-domain protein 1(JAZ1) were observed at 2h stage in both conditions of 10T2h and 50T2h. JAZ proteins as transcription repressors bind to promoter regions of jasmonate-inducible genes to block their expression [40].

In the network of time point 24h (Fig 6B and S10 Data), HSFB2B with some HSPs/chaperones were clustered. HSFB2B represses the transcription of heat shock response genes under normal temperature conditions. In contrast, under heat stress conditions, it is required for the transcription of heat stress-inducible*HSP* genes that are indispensable for acquired thermo-tolerance [41]. The expression of *HSFB2B* is necessary to attenuate harmful effect of abiotic stress on the circadian system [42], and it is also involved in resistance to biotic stress in Arabidopsis [43]. Therefore, the DEGs from the RNA-seq data constructed a strong connection between exogenous Tre and the signal pathways of both abiotic and biotic stresses.

## Discussion

Pretreating crops with natural isolated elicitors in the absence of pathogens can enhance plant resistance to diseases. Up to now, diverse elicitors are available from organisms such as bacteria, fungus, oomycete and plants. Nonetheless, different elicitor usually does not trigger same plant response. Therefore, their molecular networks need to be elucidated prior to the application of elicitor for good agriculture practice. Tre is a non-toxic disaccharide synthesized by organisms such as some bacteria, fungi, plants and invertebrate animals, and is a good candidate of elicitor against environmental stresses, especially plant diseases, as it has been more affordable and accessible due to its chemical synthesis and, when applied exogenously, is readily absorbed by plants [44]. Evidences showed that exogenous Tre reduced stomata aperture through a H_2_O_2_-dependent pathway [45, 46], and stomata movement in response to abiotic and biotic stress are regulated by redox-dependent signaling [47, 48]. It has been used as a tool to enhance plant resistances to some of abiotic and biotic stresses that affect plant growth and yield. However, exogenous Tre is more or less toxic to plant development. In Arabidopsis, 100 mM of exogenous Tre treatment results in strong inhibition of seedling growth. T6P rapidly accumulating in cytosol might mediate the signal transduction pathways of growth inhibition caused by Tre [49], which enhances starch biosynthesis and triggers redox activation of ADP-glucose pyrophosphorylase (AGPase) gene in source tissues[50]. Microarray analysis shown that Arabidopsis seedlings treated with 30 mM of had many changes on its transcripts related to metabolism, abiotic and biotic stresses, and root elongation was also inhibited significantly [21]. However, low millimolar concentrations of exogenous Tre can attenuate impairment of salt stress to Arabidopsis [51] and *Catharanthus* [52] through regulation of ionic balance, cellular redox state, cell death, and osmotic adjustment. During the process, Tre toxicity to plant was counteracted by impairment of salt stress. Furthermore, in tobacco, at low concentration (e.g. 8 mM), exogenous Tre can recover the nitrate reductase activity partially, and chlorophyll and total nitrogen content of leaves and rates of photosynthesis were increased through a long-term effect, which can be observed 1–2 weeks after the treatments [23]. In stark contrast, we did not find the expression level of any nitrate reductase genes were changed in our RNA-seq data (S7 and S8 Data). One possibility is we only focused on the molecular mechanism occurring early (in 24 h) and without any environmental stresses; and nitrate reductase genes are uniquely affected by Tre under limiting nitrogen condition. The interesting differences between our present research and Lin et al’s work [23] suggest that there might be some other mechanisms of plant responding to exogenous Tre treatment, such as performing the treatments during or after environmental stresses.Similar to this, in the present study, high concentration of Tre (50 mM) induced less number of DEGs, but showed a better protection against TMV. One possibility is, as a natural chemical compound, high concentration of Tre might act mainly as a priming agent, but not an elicitor, which means Tre can act mainly as an elicitor or a priming agent in one species in a concentration-dependent way, because it has been reported one compound can function as an elicitor or a priming agent between different species [53].

Plant pathogen is perceived by plant surface pattern recognition receptors that detect conserved microbe-specific molecules referred as to pathogen-associated molecular patterns (PAMPs). Flagellin insensitive 2 (FLS2), EF-Tu receptor (EFR) and chitin elicitor receptor kinase 1 (CERK1) are this sort of receptors that have been characterized recently referred as to pattern-recognition receptors (PRRs) involved in the specific interaction with corresponding elicitors, respectively [54–56]. The three receptors, BRASSINOSTEROID INSENSITIVE1-ASSOCIATED KINASE1 (BAK1) and BAK1-LIKE1 (BKK1) functioning as the partners of FLS2 and EFR, were all increased significantly and uniquely at 24h following 10 mM of Tre treatment (S13 Data; S5 Fig), which are strongly indicating that low concentration of exogenous Tre can effectively trigger plant-pathogen interaction pathways in tobacco leaf in a time-specific way. Activation of these receptors and their partners lead to changes in ion flux and metabolism, accumulation of ROS and hormone ethylene and MAP kinase (MPK) activation, etc [57]. Based on our RNA-seq data, the Plant-pathogen interaction pathways were enriched in both 10T2h and 10T24h. 20 DEGs from 10T2h and 61 DEGs from 10T24h were mapped to this pathway, respectively (S4 and S5 Figs). The majority (15/20 = 75%) of the 20 genes in 10T2h (S12 Data) were down-regulated. In contrast, 53/61 (around 87%) in 10T24h (S13 Data) were up-regulated, indicating that the activation of pathogen resistance genes by exogenous Tre is time-dependent. Beside those receptor like kinases mentioned above, Some cyclic nucleotide gated channels (CNGCs) proteins sub-locating on cytoplasmic membrane as the upstream components in the pathways were up-regulated significantly in the condition of 10T24h. CNGCs activates downstream of Ca2+ -mediated signal (S5 Fig; S13 Data). Functional analyses of members of this channel family have associated many of them with inward Ca^2+^ currents [58]. More and more evidence implies there is a mutual interplay between Ca^2+^- and ROS-mediated signaling pathways that might be involved in fine-tuning of intercellular and intracellular signaling networks [59]. One example is calcium acts as the prime regulatory molecule of NADPH oxidases/RBOHs that are the key player of pathogenic ROS generation in plants [60, 61]. In the model plant *Arabidopsis thaliana*, there are 10 members of RBOHs whereas nine members are present in rice plants [61]. In *Nicotiana tabacum* three members are reported [34, 35]. We measured the mRNA levels of *rbohF*, *rbohD* by qRT-PCR (Fig 1D), since they two have been proved for ROS generation in plants [34–36], and both superoxide ion and hydrogen peroxide were induced significantly at 2h and 24h after Tre treatment in tobacco leaves (Fig 1A). One *RBOH* gene in the RNA-seq data (gi|697111405|) was increased significantly at 24h after 10 mM of Tre treatment too (S5 Fig and S13 Data).

ROS are high reactive molecules, and their excessive accumulation might lead to strong damages to cell membrane system and other structures, thus originally they were deemed as detrimental byproducts during aerobic metabolism, especially when organism suffers environmental stresses. Now, it is undoubted that ROS of sub-toxic levels function as signaling molecules sensing diverse upstream signals coming from developmental processes and environmental stresses [59, 62], and subsequently transduce the signals to downstream targets as a common plant response. Different environmental stresses or stress combinations that occur in field condition often might lead to the formation of different ROS signatures, which perceived by diverse ROS sensors that activate the corresponding stress signals in plants [63–65]. For instance (S5 Fig), Ca^2+^ and ROS induce expression of pathogenesis-related (PR) genes and formation of localized cell death (LCD) at the site of infection (hypersensitive response) through activation of CDPK that was up-regulated significantly in the condition 10T24h (S13 Data); Ca^2+^ induces stomatal closure appears through calmodulin (CaM) and calmodulin-like (CML) proteins (main calcium sensors)and ROS/NO productions [66, 67]. In this study, up-regulation of CaM/CML was observed in the condition 10T24h (S5 Fig and S13 Data), and ROS (O_2_^•-^andH_2_O_2_) were accumulated in tobacco leaves after Tre treatment (Fig 1A). WRKY transcription factors, including WRKY22, 25, 29 and 33, were up-regulated in the condition 10T24h (S13 Data), and they are downstream of mitogen-activated protein kinases, MPK3/6 and MPK4. MPK4 can sense Ca^2+^-mediated signals and activate the WRKY transcription factors (S5 Fig). Although we did not observe any MPK3, MPK4 and MPK6-like genes in the 1128 DEGs, gene_23843 encodes AP2C1 protein which belongs to the PP2C-superfamily clade B and functions as a MAPK phosphatase that negatively regulates MPK4 and MPK6 [68]. In the RNA-seq data, AP2C1 was down-regulated significantly at 2h after Tre treatment independent of its concentration (S10 Data and Fig 6A), suggesting some MPKs might be activated during a particular window after Tre treatment.

HSPs, also known as molecular chaperones, play critical roles in protein correct folding and subunit assembly, translocation between cellular compartments, and targeting misfolded proteins to the proteasome in diverse normal cellular processes, and protect and stabilize proteins and membrane system against environmental stresses[69, 70], and their expressions are usually regulated by heat shock factors (HSFs) that also respond to diverse biotic and abiotic stresses. For example, it has been reported that Arabidopsis HSFA2, as a key regulator, is involved in signaling pathways of heat, high light and osmotic stress [38, 71]. Accumulated evidence have been gotten recently on redox-dependent regulation of HSFs in plants and mammalian [72, 73], further supporting the hypothesis that some oxidative stress-responsive genes are probably direct targets of oxidative stress-responsive HSFs that act as H_2_O_2_ sensors in plants [74]. It is evidenced by the report that Arabidopsis HSFA1a directly senses H_2_O_2_ via its N-terminal region from 48 to 74 amino acid residues [75]. FTHS6 encodes a plastid metalloprotease, and it regulates thermomemory in Arabidopsis through regulating HSP21 abundance [76]. Moreover, down-regulation of chloroplast FtsH Protein in TMV–infected tobacco leaves accelerates the hypersensitive reaction [77]. *GolS1* encodes a galactinol synthase and is a target of AtHSFA2 under heat stress [38, 71]. The transcription of *AtGolS1* was also induced by exogenous H_2_O_2_ in Arabidopsis [78] and other abiotic stresses such as drought, salt, or heat stress [79] and hormone ABA [80]. Its over-expression in transgenic plants promoted accumulation of galactinol, raffinose and stachyose, which resulted in enhanced resistance to abiotic stresses such as drought, salinity or cold [79, 80]. It has been reported that an Arabidopsis USP modulates ROS homeostasis under anoxic conditions [81], and one USP in tomato is a phosphorylation target of protein kinase CIPK6 and functions in oxidative stress response pathway [82]. Therefore, the 8 core genes are all in response to oxidative stress directly or indirectly, indicating that ROS are the key regulators for the DEGs triggered by exogenous Tre.

There are some important common mediators such as calcium ions between heat shock responses and defense responses [83]. Our RNA-seq data also evidenced there is a connection between components in plant-pathogen interaction pathways and HSPs triggered by exogenous Tre. As mentioned above, ROS can stimulate some HSFs directly. These HSFs as ROS sensors in turn activate expression of HSP chaperone genes and also regulate expression of ROS scavenger genes [73], such as APX2 that was up-regulated at 2h after Tre treatment (Fig 6A), which is responsible for keeping ROS homeostasis through negative feedback. Many of HSFs can specifically bind DNA sequence 5’-AGAAnnTTCT-3’ referred to as heat shock promoter elements (HSE) that are included in the promoter regions of HSPs. HSFA6B, HSFB2A and 3 HSFB2Bs were up-regulated in one or two conditions out of the four conditions (S3–S6 and S14 Data). Their orthologs in Arabidopsis or rice have strong correlations with MPK3/6 and 4, respectively, base on PPINs (STRING analysis) (S1–S3 Figs). Plant pathogen signaling pathway in KEGG shows that MAP3/4/5/6 play keys roles in the signal transductions of pathogen infection and/or attack together or alone (S4 and S5 Fig). HSFA6B has connections with eight MPKs (3,4,5,6,7,10,12 and 14) and two HSP70 proteins (S1 Fig). HSFB2A associates with four MPKs (1, 3, 5 and 11) and some HSPs (e.g. HSP 70, 80 and 90) (S2 Fig). Although in Arabidopsis, AtHSFB2B only has second shell of interactions with MPK6 (S3A Fig); in rice, HSFB2B (rice gene ID: 4346226) has correlations with three MPKs, respectively (rice gene ID: 4344698, 4341956 and 4349225) (S3B Fig). It has been reported MAP kinase kinase 5 (MPPK5) might be a direct target of HSFB1/HSFB2B based on microarray analysis [43]. In addition, HSFA4A confers plant salt resistance and is regulated by oxidative stress. It has a physical interaction with MPK3 and MPK6 and is phosphorylated by the two mitogen-activated protein kinases [84]. Arabidopsis HSFA2 is also regulated by MPK6-targeted phosphorylation during heat stress response [85]. The heat shock transcription factor HSFB2B is strongly responsive to photorespiratory H2O2 [86]. Some HSPs as HSFs targets are not only involved in heat stress, but also function in microbial pathogenesis such as HSP70 [87]. In addition, HSP90 gets involved in innate immune responses in tobacco through interacting with SGT1 and RAR1 they are critical signaling components in resistance (R) gene-mediated plant resistance responses [88], and HSP90 is also involved in resistance to TMV through interacting with resistance protein N [88, 89]. Besides the top ten KEGG pathway in which the total DEGs were mapped, we found that some other pathways were uniquely enriched in the DEGs triggered by 10mM of Tre, but not in those triggered by 50 mM of Tre, such as ATP-binding cassette transporters (ABC transporters). In the RNA-seq data, 8 ABC transporter genes were up-regulated and 1 gene was down-regulated (Fig 5; S7 Data). The integral membrane proteins transporters are widespread in all living organisms and can be grouped into exporters and importers. They are not only required for organ growth, plant nutrition and plant development, but also respond to both abiotic and biotic stresses [90].

**Fig 7:**
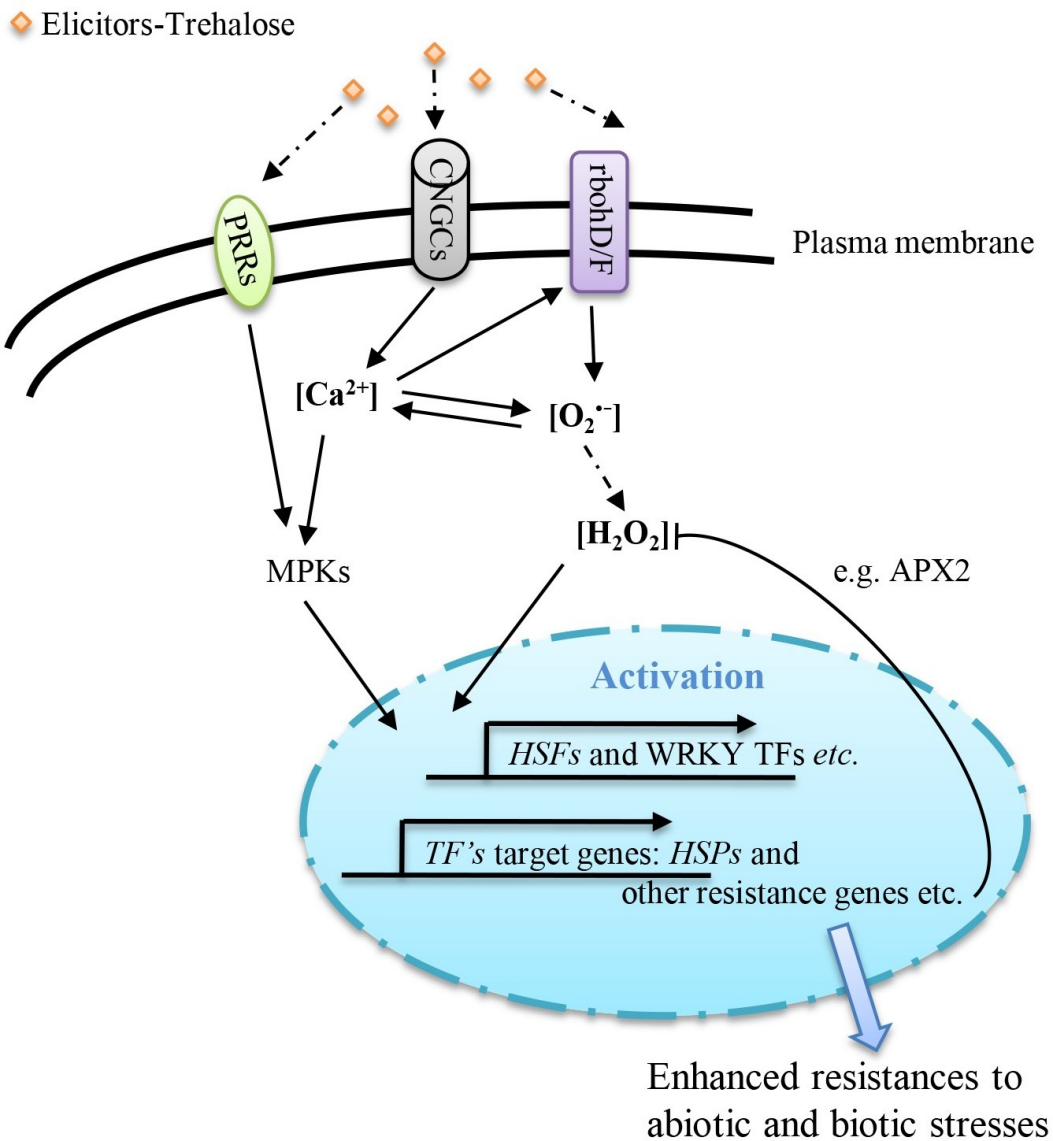
Core signaling in response to exogenous trehalose treatment. *Abbreviations*:PRRs—Pattern-recognition receptors; FLS2—Flagellin insensitive 2; EFR—EF-Tu receptor; CERK1—Chitin elicitor receptor kinase 1;CNGCs—Cyclic nucleotide gated channels; O_2_^•-^—superoxide ion; H_2_O_2_—hydrogen peroxide; MPKs—MAP kinases; APX2—Ascorbate peroxidase 2; HSFs—Heat shock factors; TF—Transcription factor; HSPs—Heat shock proteins. Dotted arrows represent the unknown pathways.

In summary, exogenous Tre as an elicitor molecule recognized by tobacco cells, and trigger intracellular pathways related to defense, via the mutual interplay between calcium and ROS signaling systems (Fig 7).

## Conclusion

This study provides new insight into the molecular basis of Nicotiana tobacum leaves in response to exogenous Tre treatment by comprehensive analysis of gene expression profiles. Our results also showed that tobacco leaf treatment with higher concentration of Tre (50 mM) conferred the plant better disease resistance to TMV. In contrast, a lower concentration of Tre (10 mM) triggered more genes differentially expressed, which are responsive to Ca^2+^ and ROS-mediated signaling, in noninfectious context. However we didn't investigate any priming effect of Tre which could be involved in protection induced against TMV. Priming is characterized by a faster and/or stronger activation of cellular and molecular defenses responses that are induced following a pathogenic attack. Such a priming effect might explain the better protection obtained with 50mM Tre pretreatment before infection with TMV. For further researches, we will investigate this possibility, and characterize the molecular functions of the DEGs triggered by exogenous Tre via transgenic approaches.

## Supporting information

S1 Data The statistical data of Table 1. (XLSX)

S2 Data List of primers for qRT-PCR. (XLSX)

S3 Data DEGs expressed in the condition of 10T2h. (XLSX)

S4 Data DEGs expressed in the condition of 10T24h. (XLSX)

S5 Data DEGs expressed in the condition of 50T2h. (XLSX)

S6 Data DEGs expressed in the condition of 50T24h. (XLSX)

S7 Data DEGs regulated by 10 mM of trehalose uniquely. (XLSX)

S8 Data DEGs regulated by 50 mM of trehalose uniquely. (XLSX)

S9 Data Overlapping DEGs regulated by all conditions. (XLSX)

S10 Data Overlapping DEGs regulated by 10T2h and 50T2h. (XLSX)

S11 Data Overlapping DEGs regulated by 10T24h and 50T24h. (XLSX)

S12 Data DEGs of 10T2h in plant pathogen pathway in KEGG. (XLSX)

S13 Data DEGs of 10T24h in plant pathogen pathway in KEGG. (XLSX)

S14 Data HSFs induced by exogenous trehalose. (XLSX)

S1 Fig The PPIN of HSFA6B and MPKs. (TIF)

S2 Fig The PPIN of HSFB2A and MPKs. (TIF)

S3 Fig The PPIN of HSFB2B and MPKs. (TIF)

S4 Fig KEGG map of 10T2h DEGs in plant pathogen pathway. (TIF)

S5 Fig KEGG map of 10T24h DEGs in plant pathogen pathway. (TIF)
