# Supplementary figures and images for: Physiological and transcriptomic analyses reveal the molecular networks of responses induced by exogenous trehalose in plant
Source: PLoS One. 2019 May 22;14(5):e0217204. doi: 10.1371/journal.pone.0217204 (PMC6530874; doi:10.1371/journal.pone.0217204)

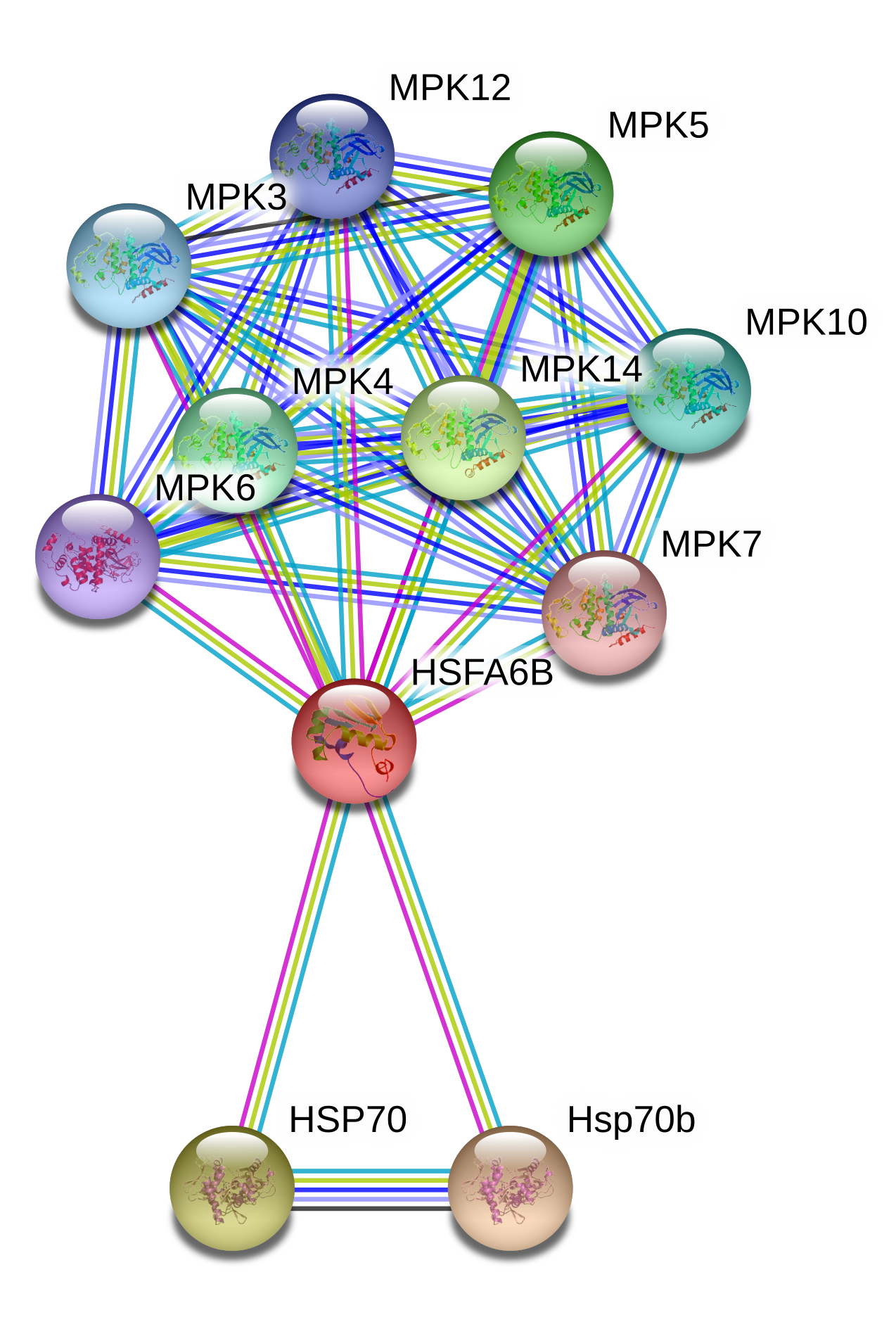

Supplement: S1 Fig — (TIF) [file pone.0217204.s015.tif]

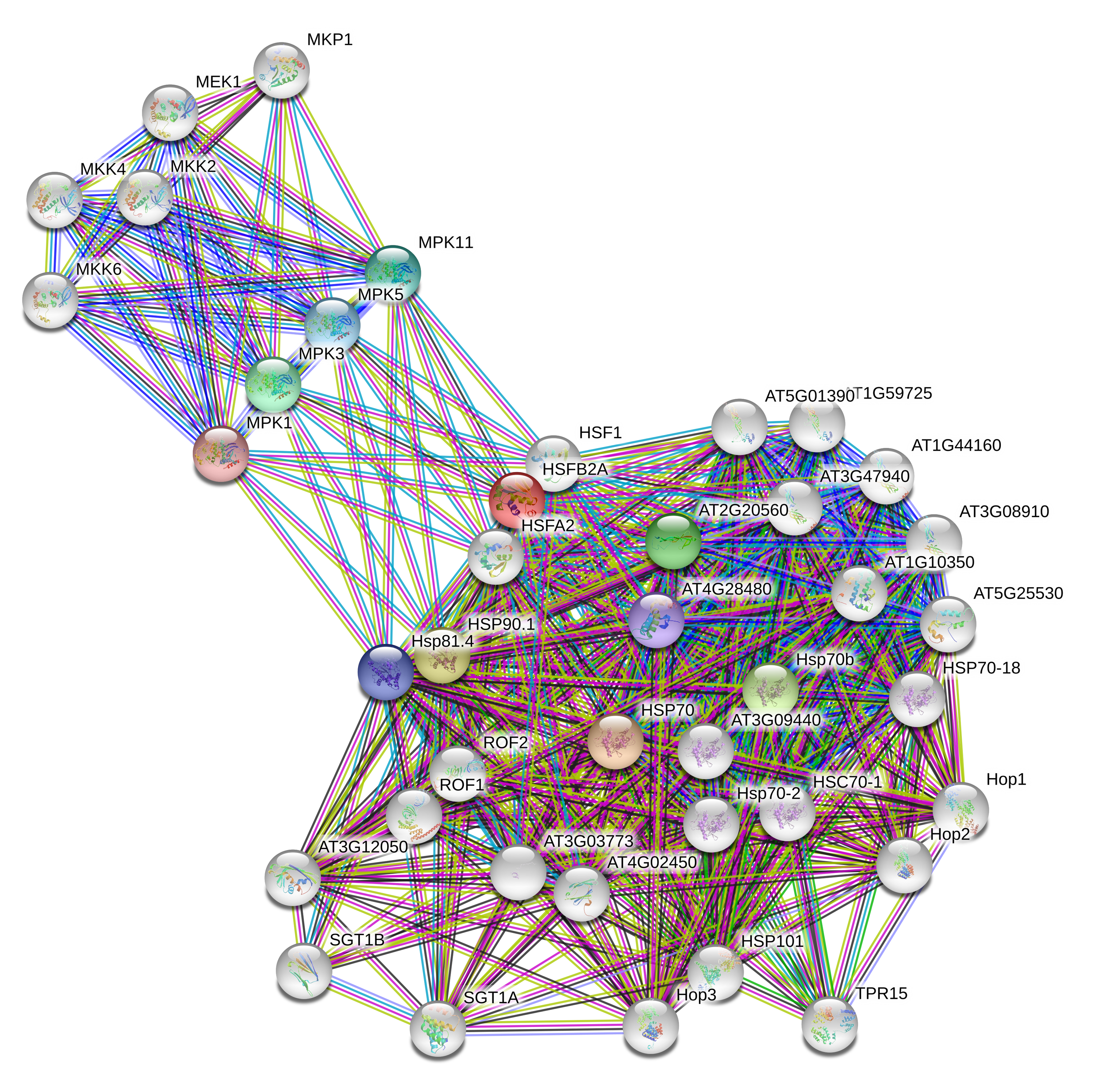

Supplement: S2 Fig — (TIF) [file pone.0217204.s016.tif]

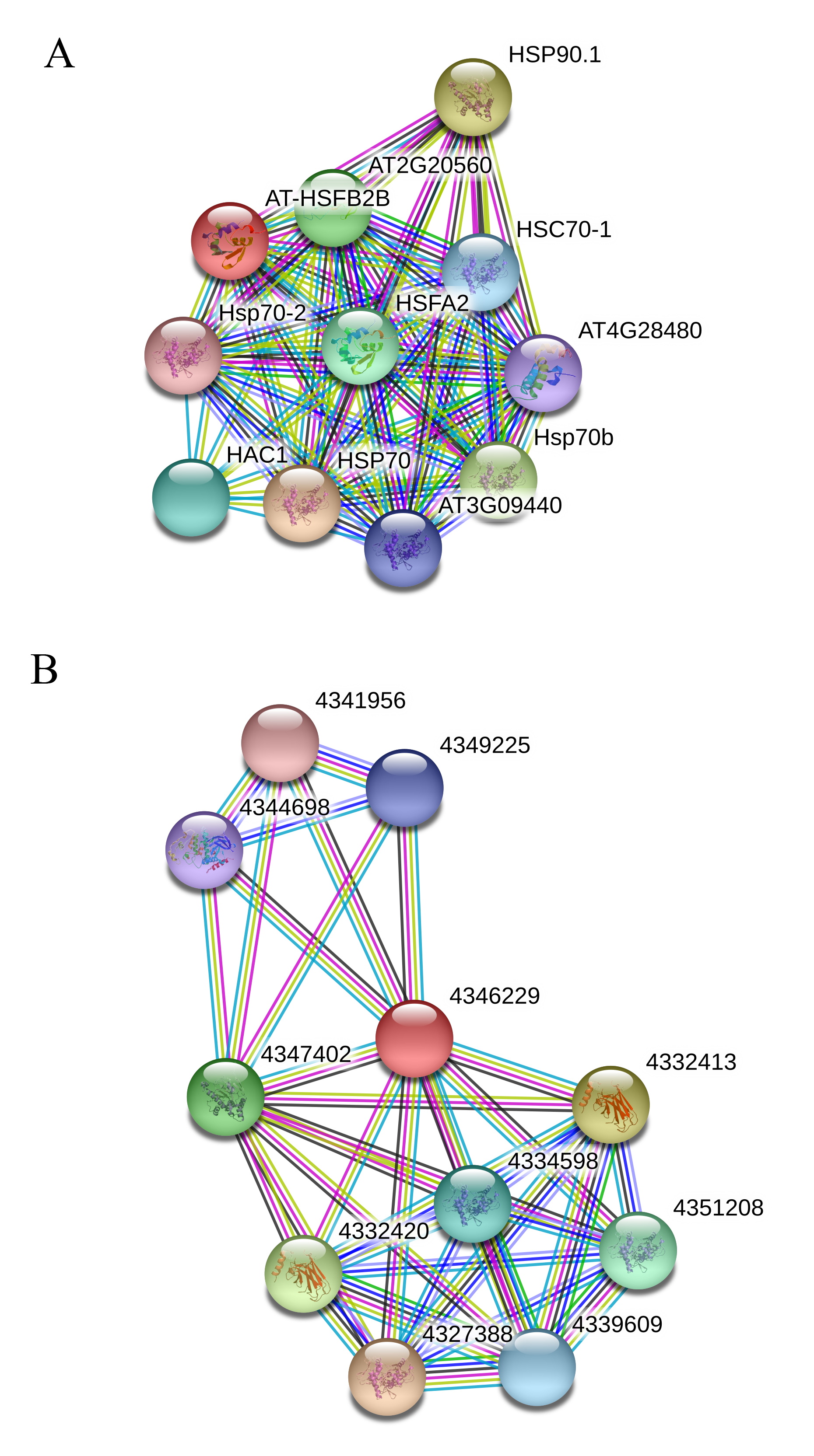

Supplement: S3 Fig — (TIF) [file pone.0217204.s017.tif]

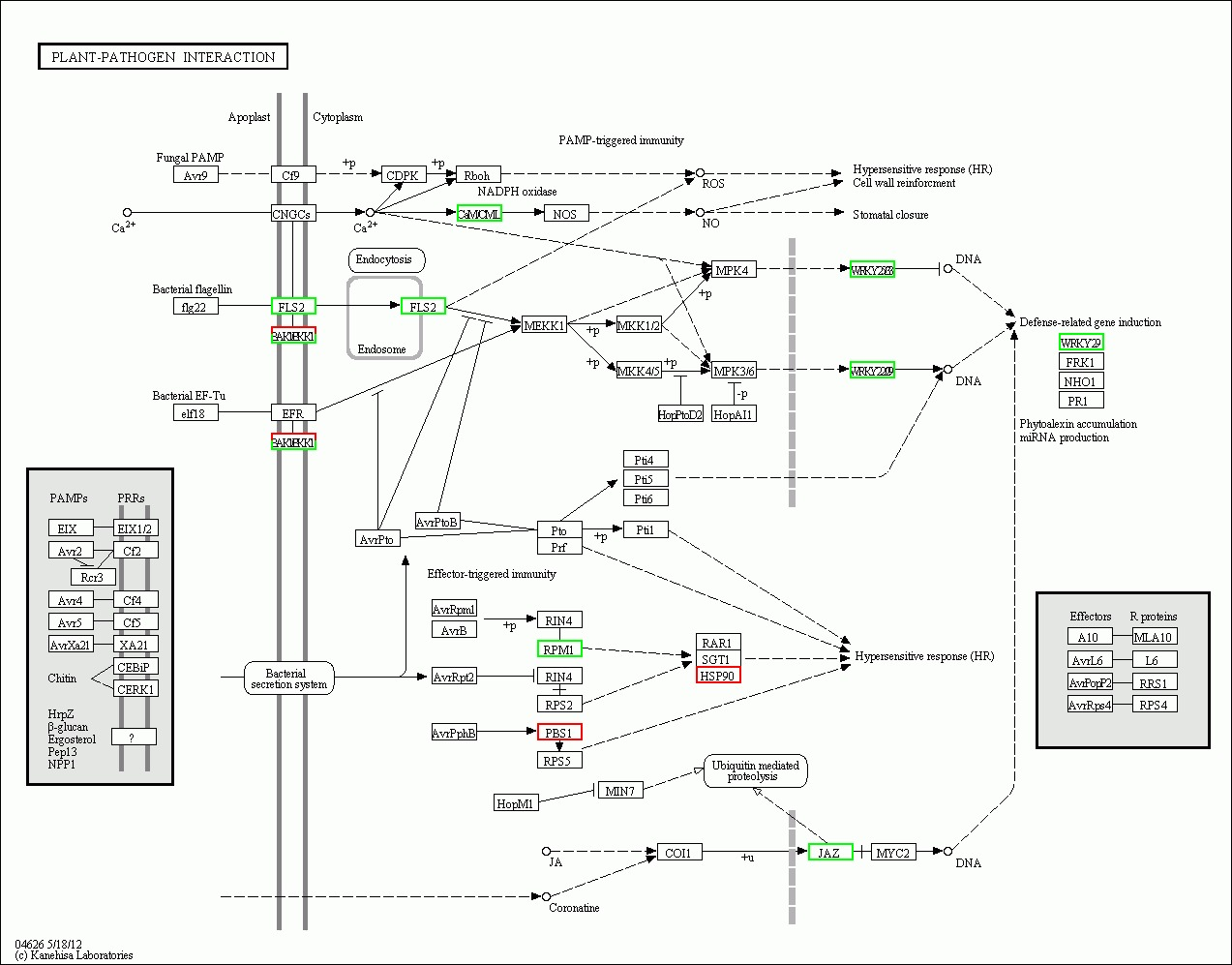

Supplement: S4 Fig — (TIF) [file pone.0217204.s018.tif]

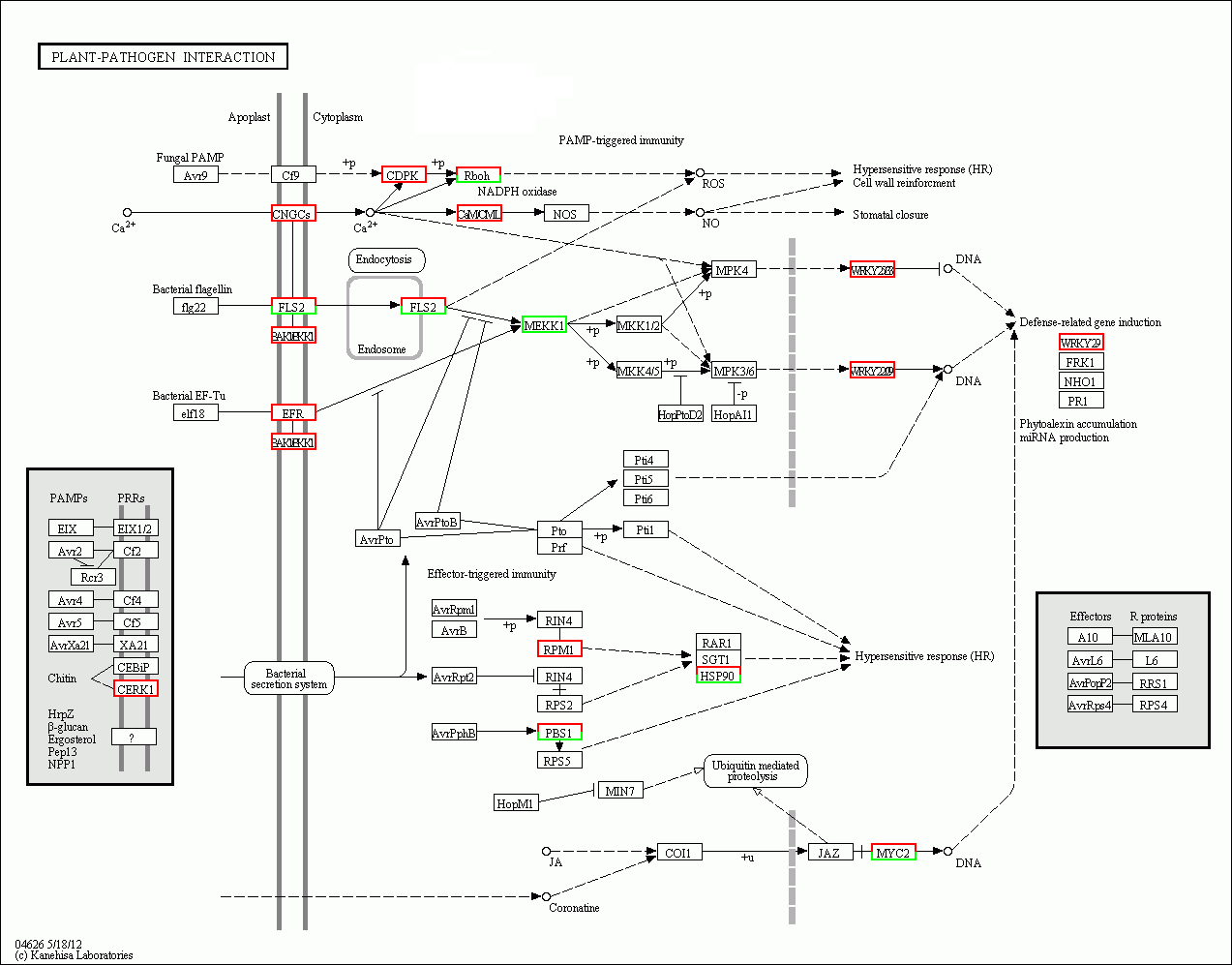

Supplement: S5 Fig — (TIF) [file pone.0217204.s019.tif]
